# Multiple-bead assay for the differential serodiagnosis of neglected human cestodiases: Neurocysticercosis and cystic echinococcosis

**DOI:** 10.1371/journal.pntd.0010109

**Published:** 2022-01-14

**Authors:** Ana Hernández-González, Belén González-Bertolín, Laura Urrea, Agnes Fleury, Elizabeth Ferrer, Mar Siles-Lucas, Francesca Tamarozzi, Maria J. Perteguer

**Affiliations:** 1 Laboratorio de Helmintos, Centro Nacional de Microbiología (CNM), Instituto de Salud Carlos III, Majadahonda, Madrid, Spain; 2 Departamento de Microbiología y Parasitología, Facultad de Farmacia, Universidad Complutense de Madrid, Madrid, Spain; 3 Instituto de Investigaciones Biomédicas, Universidad Nacional Autónoma de México (UNAM); México/Instituto Nacional de Neurología and Neurocirugía, Mexico City, Mexico; 4 Instituto de Investigaciones Biomédicas "Dr. Francisco J. Triana Alonso" (BIOMED), Departamento de Parasitología, Facultad de Ciencias de la Salud, Universidad de Carabobo Sede Aragua, Maracay, estado Aragua, Venezuela; 5 Instituto de Recursos Naturales and Agrobiología de Salamanca, Consejo Superior de Investigaciones Científicas (IRNASA-CSIC), Salamanca, Spain; 6 Department of Infectious-Tropical Diseases and Microbiology, IRCCS Sacro Cuore Don Calabria Hospital, Negrar, Verona, Italy; National Institute for Infectious Diseases (L. Spallanzani), ITALY

## Abstract

**Background:**

Neurocysticercosis (NCC), and cystic echinococcosis (CE) are two neglected diseases caused by cestodes, co-endemic in many areas of the world. Imaging studies and serological tests are used in the diagnosis of both parasitic diseases, but cross-reactions may confound the results of the latter. The novel multiplex bead-based assay with recombinant antigens has been reported to increases the diagnostic accuracy of serological techniques.

**Methodology:**

We set-up an immunoassay based on the multiplex bead-based platform (MBA), using the rT24H (against *Cysticercus cellulosae*, causing cysticercosis) and r2B2t (against *Echinococcus granulosus sensu lato*, causing CE) recombinant antigens, for simultaneous and differential diagnosis of these infections. The antigens were tested on 356 sera from 151 patients with CE, 126 patients with NCC, and 79 individuals negative for both diseases. Specificity was calculated including sera from healthy donors, other neurological diseases and the respective NCC or CE sera counterpart. The diagnostic accuracy of this assay was compared with two commercial ELISA tests, Novalisa and Ridascreen, widely used in the routine diagnosis of cysticercosis and CE, respectively.

**Main findings:**

For the diagnosis of NCC, sensitivity ranged from 57.94–63.49% for the rT24H-MBA, and 40.48–46.03% for Novalisa ELISA depending on exclusion or inclusion of sera having equivocal results on ELISA from the analysis; specificities ranged from 90.87–91.30% and 70.43–76.96%, respectively. AUC values of the ROC curve were 0.783 (rT24H) and 0.619 (Novalisa) (p-value < 0.001). For the diagnosis of CE, the sensitivity of the r2B2t-MBA ranged from 68.87–69.77% and of Ridascreen ELISA from 50.00–57.62%; specificities from 92.47–92.68% and from 74.15–80.98%, respectively. AUC values were 0.717 and 0.760, respectively.

**Conclusions/Significance:**

Overall, the recombinant antigens tested with the bead-based technology showed better diagnostic accuracy than the commercial assays, particularly for the diagnosis of NCC. The possibility of testing the same serum sample simultaneously for the presence of antibodies against both antigens is an added value particularly in seroprevalence studies for cysticercosis linked to control programs in endemic areas where these two parasites coexist.

## Author summary

Cysticercosis and cystic echinococcosis are caused by infection with the larval stages of *Taenia solium* and *Echinococcus granulosus sensu lato*, respectively, and their distribution largely overlaps in many areas of the world. Currently, the WHO is calling for the need to map endemic areas for cysticercosis, where control programs should be applied. Serosurveys may serve this scope, but cross-reactivities may be an issue. This study evaluated the accuracy of two recombinant antigens (rT24H and r2B2t) specific for each parasite used together applying a novel multiple bead technology assay (MBA), and compared the results with two commercial ELISA tests for the diagnosis of these infections. We found that this method, although not 100% specific, provides better results than those obtained with the commercially available tests, with the added advantage of testing simultaneously for both infections using just one sample, and could prove an efficient and rapid support for serological screening studies to map areas of cysticercosis transmission. Importantly, this novel method based on MBA technology is open and flexible, allowing continuous improvement due to its ability to simultaneously introduce new recombinant antigens as they are discovered/defined.

## Introduction

Cysticercosis (CC) and cystic echinococcosis (CE) are zoonoses included in the list of neglected tropical diseases. Both parasitic infections are caused by the larval stages (metacestodes) of cestodes belonging to the family Taeniidae, *Taenia solium*, and *Echinococcus granulosus sensu lato*, respectively, which develop in host tissues. CC is prevalent in Latin-America, Asia and sub-Saharan Africa, although it is currently diagnosed also in regions where it was not previously endemic because of travel and migration [1,2]. CE has a cosmopolitan distribution, and is especially prevalent in pastoral communities in Western China, Central Asia, Mediterranean and Eastern European countries, Eastern Africa, and South America [3,4]. CC and CE, the former especially when involving the central nervous system, have significant public health and economic impact [5,6], ranking first and second, respectively, among food-borne parasites worldwide enlisted by the Food and Agriculture Organization of the United Nations [7]. The global burden of human CC and CE has been estimated in 2.5–5 million and nearly 1 million disability-adjusted life years, respectively [8,9].

Most human infections caused by *T*. *solium* cysticerci affect the central nervous system, causing neurocysticercosis (NCC). This disease is one of the most frequent neuroparasitoses, as well as one of the primary causes of preventable seizures worldwide [10]. CE is caused by *Echicocococcus granulossus* s.l. metacestodes (cysts), which develop most commonly in the liver (about 70% of the cases) followed by the lungs. Clinical manifestations range from asymptomatic infection, which can last several years, to severe conditions. Symptoms are the consequence of cyst growth, and depend on the location of cyst and compression caused on neighbouring structures, that may lead to life-threatening complications such as cyst rupture [11].

Imaging studies are the most relevant diagnostic procedures for NCC and CE [12], supported by clinical and epidemiological criteria [13,14]. In the case of NCC, although serological techniques are included in the diagnostic criteria, currently available seroassays have non-optimal sensitivities and specificities [15–18]. On the one hand, for both infections, sensitivity is influenced by many factors such as localization, stage of development, and number of metacestodes. On the other hand, specificity may be particularly poor due to cross-reactivity between the two as well as other parasites, making the interpretation of seroassays difficult in co-endemic areas. The impossibility to rely on serosurveys for an accurate diagnosis of infection with these parasites, together with the slow and most often asymptomatic progression of these diseases, are also partly responsible for the underestimation of their prevalence, as only a fraction of cases is diagnosed [4,19,20]. It is therefore evident that any improvement of serological techniques for the diagnosis of these infections would better support their epidemiological mapping, diagnosis, and control, as flagged by the WHO roadmap for 2030 [21].

The aim of this study was to evaluate the diagnostic accuracy of two recombinant antigens (rT24H for the diagnosis of CC and r2B2t for CE), previously evaluated separately [22–24], for the serodiagnosis of CC hot-spots in CE co-endemic areas in a combined diagnostic system using a multi-analyte multiplex bead-based assay (MBA) platform.

## Materials and methods

### Ethics statement

All serum samples from patients with CE were obtained from patients attended at the division of Infectious and Tropical Diseases, San Matteo Hospital Foundation, Pavia, Italy and all of them were anonymized. The use and transfer of stocked human sera leftovers from routine analyses carried out in San Matteo Hospital Foundation, Pavia, Italy, was approved by the Ethics Committee of IRCCS San Matteo Hospital Foundation, Pavia, Italy (Acceptance Report 2015041 of 06/07/2015). Serum samples from patients with NCC, other neurological diseases, and healthy individuals were obtained from patients followed at the Instituto Nacional de Neurología y Neurocirugía (INNN), México. Written informed consent was obtained from all patients for the storage and use for research purposes of their serum. All sera were stored in the repository C.0003989- ISCIII Biobank.

### Serum samples

The study was performed with 356 serum samples: 126 from patients with NCC (Table 1, Set 1), 151 from patients with CE (Table 1, Set 2), and 79 negative controls (Table 1, Set 3).

**Table 1:** Sample collection. Characteristics and number of tested serum samples.

Sera categories	N
**NCC samples (set 1)** *	
Viable cysts (active)	48
Non-viable cysts (inactive)	63
Unknown data	15
Total NCC	126
**CE samples (set 2)** **	
CE1-CE3b (active)	**77**
CE1	4
CE2	2
CE3a	19
CE3b	52
CE4-CE5 (inactive)	**74**
CE4	54
CE5	20
Total CE	**151**
**Samples negative for NCC and CE (set 3)**	79
**Total number of sera**	356

CE: cystic echinococcosis; NCC: neurocysticercosis; N: number of sera.

*25/126 (20.6%) in extraparenchimal localization.

**145/151 (96%) localized in the liver; 106/151 (70.2%) untreated.

The diagnosis of abdominal CE was based on pathognomonic signs on ultrasonography (US, reference standard), and CE cyst were staged according on the WHO Informal Working Group on Echinococcosis (WHO-IWGE) classification [25]. CE cysts were further grouped into active (CE1-CE3b) and inactive (CE4-CE5) for analysis. When multiple cysts were present, the patient was classified according to the cyst stage known to be most associated with positive serology, based on the known relation between hepatic cyst stage and sensitivity of serology, with the highest values obtained in the presence of CE2, CE3a and CE3b active cyst stages, and the lowest in the presence of the inactive cyst stages [26].

All NCC patients had a confirmed diagnosis based on validated diagnostic criteria [27]. Based on the radiological appearance, patients were divided into having active (viable cysts) or inactive (non-viable cysts) NCC.

Additionally, 79 human serum samples from uninfected subjects (Table 1, Set 3) were collected: 49 from healthy blood donors without clinical symptoms of NCC nor of CE, who fulfilled the requirements for blood donation, including absence of blood-transmitted infectious diseases,15 from healthy Mexican subjects (healthy controls from INNN without neurological symptoms nor CE), and 15 from patients with other neurological diseases including optic neuritis (n = 6), multiple sclerosis (n = 3), aneurysm (n = 1), brain tumours (n = 2), spastic paraparesis (n = 1), cerebrospinal fluid fistula (n = 1) and chronic hydrocephalus (n = 1) who may be clinically confused with NCC. Serological assays with lentil-lectin glycoprotein enzyme-linked immunoelectrotransfer blot test (LLGP-EITB) and CE-ELISA based on hydatid fluid were also performed for all uninfected control samples, with negative results.

### T24H recombinant antigen (rT24H)

**Fig 1:**
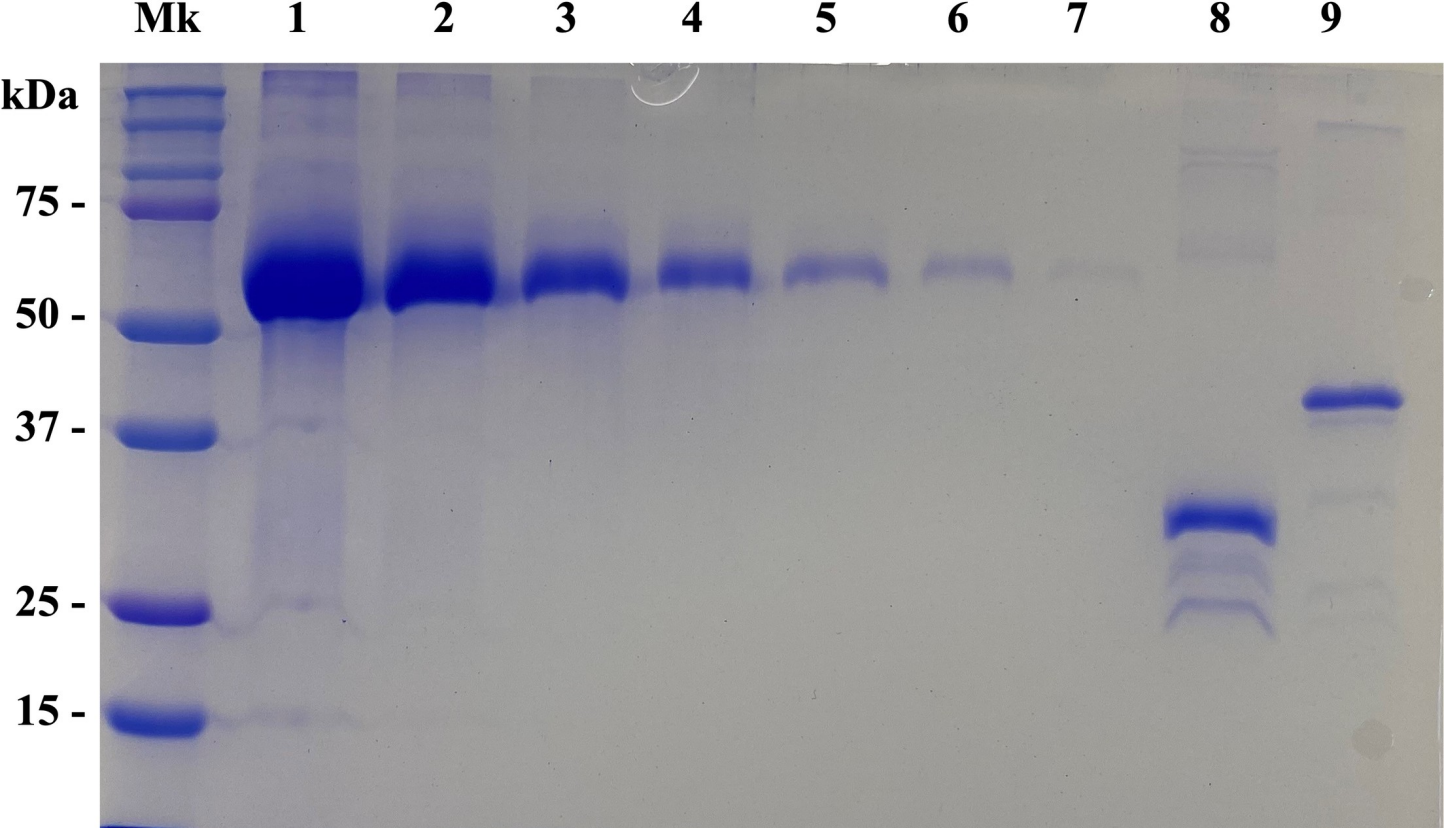
Integrity and concentration of the recombinant proteins. Lane Mk: Biorad Precision plus protein dual color marker standards. Lanes 1–7: Bovine serum albumin (BSA) protein standard curve in decreasing concentrations. Lane 1: 1 mg /mL. Lane 2: 0.5 mg /mL. Lane 3: 0.25 mg /mL. Lane 4:0.125 mg /mL Lane 5: 0.062 mg /mL. Lane 6: 0.031 mg /mL Lane 7: 0.015 mg /mL. Lane 8: 1/20 dilution of recombinant protein T24H. Lane 9: undiluted recombinant protein 2B2t.

*Escherichia coli* BL21 competent cells were transformed with the pGEX-6P-1 vector (Cytiva, MA, USA) containing the rT24H sequence (GenBank: AY211879.1) and 18 additional nucleotides at the C-terminus end corresponding to six histidine (His) residues. Expression conditions and subsequent purification of the GST-fused rT24H antigen (with the poly-His tag at the C-terminus) are described in Hernández-González et al. (2017) [28]. Integrity, purity and molecular weight were assessed by SDS-PAGE (12%); protein concentration was calculated with the Pierce BCA Protein Assay Kit (ThermoScientific, Pierce, IL, USA) and with a bovine serum albumin (BSA) protein standard curve (Fig 1). The recombinant protein was stored at -80°C until use.

### 2B2t recombinant antigen (r2B2t)

The 2B2t sequence (Hernández-González et al., 2012) [24] was subcloned into the pGEX-6P-1 expression vector (Cytiva, MA, USA) and a poly-His tag was added at the C-terminus. This construction was used to transform BL21-CodonPlus-RIL *Escherichia coli* competent cells (Agilent Technologies, Santa Clara, CA, USA). Protein expression was induced by adding 0.1 mM isopropyl-β-D-thiogalactopyranoside (IPTG, Sigma-Aldrich, Saint Louis, MO, USA) at 37°C and shaking (220 rpm) for three hours. A two-step affinity chromatography was performed for protein purification using two different sepharose resins. In the first step, GST-2B2t-poly-His recombinant protein was eluted from a Glutathione Sepharose 4B column (Cytiva, MA, USA) after incubation with L- Glutathione reduced (Sigma-Aldrich, St. Louis, MO, USA). Next, a second purification was performed on a Ni Sepharose 6 Fast Flow column (Cytiva, MA, USA) using 500 mM imidazole (Sigma-Aldrich, St. Louis, MO, USA) for elution. After dialysis against PBS, the purity, integrity and molecular weight of the protein were verified by SDS-PAGE (12%). The concentration was calculated with the Pierce BCA Protein Assay Kit (ThermoScientific, Pierce, IL, USA) and with a BSA protein standard curve (Fig 1). The recombinant protein was stored at -80°C until further usage.

### Multiplex bead-based fluorescent assay

Coupling of recombinant proteins to the surface of the magnetic beads (Bio-Plex Magnetic COOH Beads, Bio-Rad, Hercules, CA, USA) was performed as per Hernández-González et al. (2017) [28]. The rT24H protein was coupled at a concentration of 1 μg/1.25*10E^6^. For the r2B2t protein, a range of concentrations (25 μg/1.25*10E^6^ beads to 2 μg/1.25*10E6 beads) was tested to determine the optimal conditions. rT24H and r2B2t were coupled to different beads (each with their own reference code, MC10072-01 and MC10035-01, respectively) and assayed in the same well.

MagPlex immunoassays were carried out on black flat bottom 96-well plates (Costar, Fisher Scientific, Rockford, IL, USA) as described by Anderson et al. [29] with an additional pre-incubation step to improve signal detection. Serum samples were diluted 1:100 in 0.3% Tween 20-PBS containing 5% skimmed milk and a 5% *E*. *coli* lysate (from BL21 CodonPlus RIL cells) that were then incubated at 37°C for 60 minutes with stirring. The *E*. *coli* lysate was obtained as per Crestani et al. [30]. Samples were tested in duplicate as follows: 50 μl of diluted sera were added to each well together with 50 μl of 0.3% Tween 20-PBS containing 5% skimmed milk, and 2500 r2B2t- and 2500 rT24H-coupled microspheres and left to incubate on a plate shaker for 30 minutes at room temperature. Next, the plates were washed twice with Biotek Magnetic Washer ELX50 and 50 μl of a conjugated biotinylated anti-human IgG (Southern Biotech, Birmingham, AL, USA) were added to each well diluted 1:200 in PBS, 1% bovine serum albumin (BSA), and 0.05% NaN_3_. Plates were incubated and washed as described above. Next, 50 μl of streptavidin, R-phycoerythrin conjugate (Invitrogen, Carlsbad, CA, USA) diluted 1:250 in PBS, 1% BSA, 0.05% NaN_3_ were added. After a last cycle of incubation and washes, 100 μl of PBS, 1% BSA, 0.05% NaN_3_ were added into the beads-containing wells. Fluorescence intensity values for the two types of coupled beads, in each well, were read and analysed using the BioPlex manager software, version 5.0.0.531 (BioRad, Hercules, CA, USA) and a Luminex 200 platform (BioRad, Hercules, CA, USA). The reader was set to read a minimum of 100 beads with a unique fluorescent signature per region and the output was the median fluorescence intensity (MFI) per sample. MFI obtained for each sample and antigen in MBA were transformed into a serological index (MBA-SI) as follows:

$$Serological\;index=\frac{MFI\;sample–MFI\;negative\;control}{MFI\;positive\;control–MFI\;negative\;control}$$ (1)

### ELISA kits

The same sera were tested using two commercially available ELISA kits: the Novalisa *Taenia solium* IgG (NovaTec Immundiagnostica GmbH, Dietzenbach, Germany) and the Ridascreen Echinococcus IgG (R-Biopharm AG, Darmstadt, Germany). Assays were carried out according to the manufacturer’s instructions. For the Novalisa test, serum index (ELISA-SI) were interpreted following manufacture’s instructions, taking into account that it generates positives, negatives and equivocal results.

### Assays controls and statistical analysis

In addition to the test panel of sera (Table 1), MBA plates were run with the inclusion of other previously evaluated sera, used as intra- and inter-MBAs controls, to ensure repeatability between plates and detection of low positives. A single pool of negative human sera, one medium-reactive positive serum to NCC, one medium-reactive positive serum to CE, and a series of dilutions from the above two positive sera were used. NCC and CE medium-reactive sera were each obtained by the dilution of a confirmed patient serum with a pool of negative sera from healthy donors. The NCC confirmed serum was donated by Dr. Handali from Centers for Disease Control and Prevention (CDC) Division of Parasitic Diseases and Malaria (Atlanta, USA). The CE confirmed serum was obtained from a patient with confirmed CE from Hospital Universitario La Paz (Madrid, Spain).

To compare the performances of MBA-rT24H with Novalisa *Taenia solium* IgG kit, and of MBA-r2B2t with the Ridascreen *Echinococcus* IgG kit, results found in the grey zone/equivocal as defined by the ELISA assays were either discarded from the statistical analysis, or considered positive or considered negative.

Serological index values were used to construct receiver operating characteristic (ROC) curves to reach two outcomes: (i) determine cut-off values, sensitivity, and specificity for each antigen in the MBA and (ii) compare their performance with the commercial ELISA kits. Specificity was calculated considering all sera (healthy donors, neurological disease patients and the respective NCC or CE sera panel counterpart). For the ELISA commercial assays, ELISA-SI and NovaTec Units (NTU) values were used to calculate the corresponding ROC curves to compare the different tests. Results were analysed using the non-parametric approach proposed by DeLong et al. For our study, due to the correlated nature of data, DeLong’s empirical AUC approach was considered more appropriate than the binomial AUC, which requires strong normality assumptions. DeLong non-parametric approach is applied in two or more empirical curves, which tests have been performed on the same individuals [31]. The Chi-square test was used to calculate p-values and statistical significance was set at p<0.05. Data were analysed using SPSS (Version 22) and EPIDAT (Version 3.1); conservative confidence intervals were calculated using normal approximation [32].

## Results

### Recombinant antigens (rT24H and r2B2t)

Recombinant rT24H and 2B2t proteins were expressed and purified as described above. Expected molecular weights for the proteins were confirmed: 36 kDa for rT24H and 45.6 kDa for 2B2t. Final concentrations were 2.5 mg/mL and 0.1 mg/mL, respectively **(Fig 1).**

### Multiplex bead-based fluorescent assay

**Fig 2:**
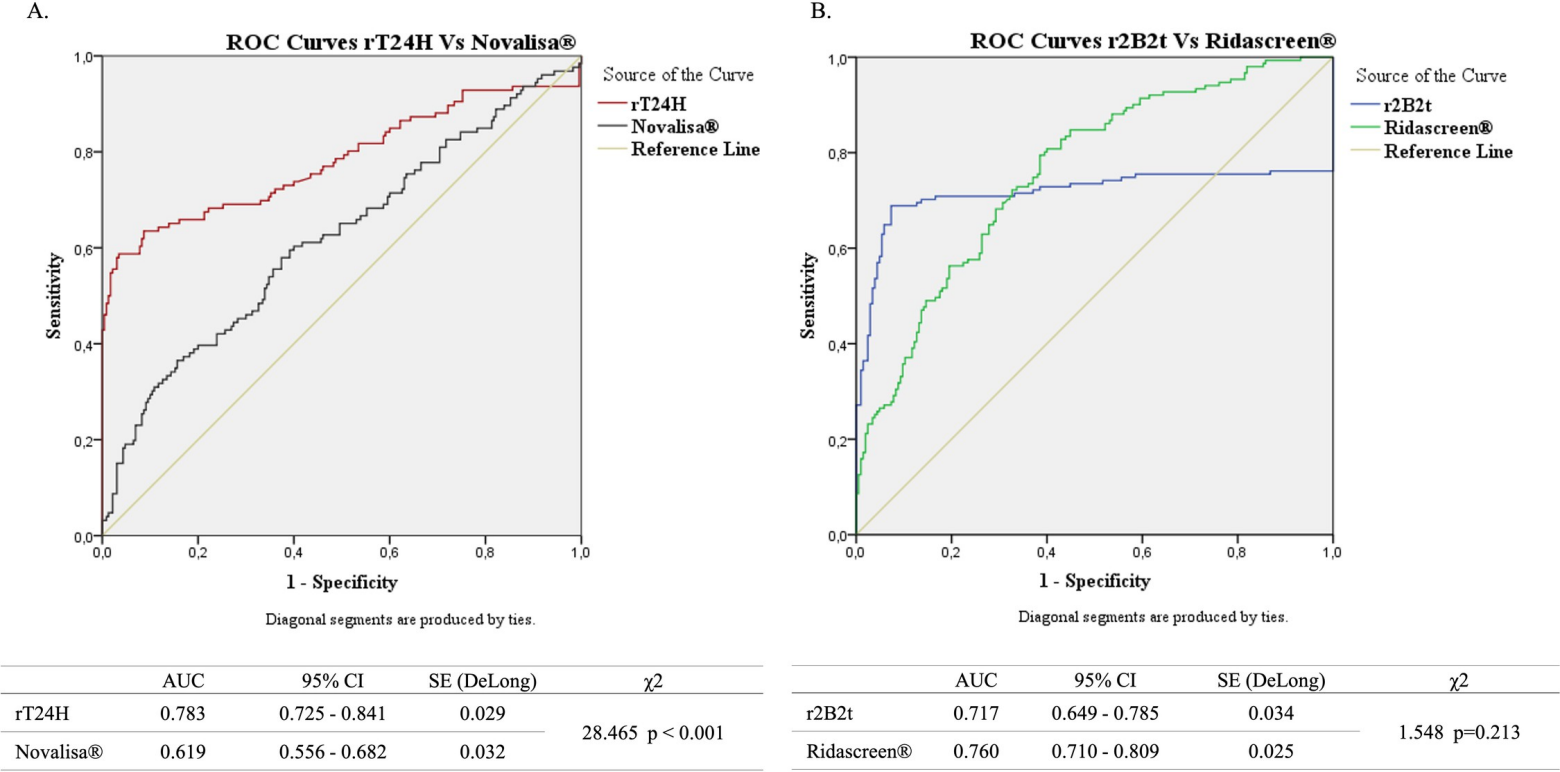
ROC curves analysis of different tests. A.ROC curves analysis showing comparison of rT24H (MBA) and Novalisa (ELISA) with all serum samples sets. B.ROC curves analysis showing comparison of r2B2t (MBA) and Ridascreen (ELISA) with all serum samples sets. Abbreviations: χ^2^:Chi-Square; SE: Standard error; CI: confidence interval; ROC: Receiver Operating Characteristic; AUC: Area under the ROC curve.

Coupling of the rT24H recombinant protein occurred at a concentration of 1μg/1.25*10E6 beads. The optimal cut-off value for the MBA-SI was established at 4.23 based on ROC curve analysis. The sensitivity obtained with this cut-off for the diagnosis of NCC was 63.49%; specificity was 100% with non-CE sera; a specificity of 91.3% was obtained when CE-positive sera were included (Table 2). The Area Under the Curve (AUC) was 0.783 (**Fig 2A**).

**Table 2:** Diagnostic test accuracy of rT24H and r2B2t in MBA.

		Recombinant antigen T24H				
Sera categories	N	(+/-)	Sensitivity (%)	95% CI	Specificity (%)	95% CI
**Positive NCC (set 1)**	**126**	**80/46**	**63.49**	**54.69–72.30**	**-**	**-**
Active NCC	48	37/11	77.08	64.15–90.02	-	-
Inactive NCC	63	30/33	47.62	34.49–60.75	-	-
**Negative** **(sets 3 and 2)**	**230**	**20/210**	**-**	**-**	**91.3**	**87.45–95.16**
Healthy (set 3)	79	0/79	-	-	100	99.37–100
CE (set 2)	151	20/131	-	-	86.75	81.02–92.49
		Recombinant antigen 2B2t				
Sera categories	N	**(+/-)**	Sensitivity (%)	95% CI	Specificity (%)	95% CI
**Positive CE** **(set 2)**	**151**	**104/47**	**68.87**	**61.16–76.59**	**-**	**-**
CE1-CE3b	77	59/18	76.62	66.52–86.73	-	-
CE4-CE5	74	45/29	60.81	49.01–72.61	-	-
**Negative** **(sets 3 and 1)**	**205**	**15/190**	**-**	**-**	**92.68**	**88.87–96.49**
Healthy (set 3)	79	0/79	-	-	100	99.37–100
NCC (set 1)	126	15/111	-	-	88.1	82.04–94.15

Abbreviations: CE: Cystic echinococcosis NCC: Neurocysticercosis N: number of sera CI: confidence interval

Regarding the recombinant r2B2t protein, after different antigen concentrations were tested, 12.5 μg/1.25*10E6 beads and a cut-off value of 4.07 were chosen. Sensitivity was 68.87% for CE, while specificity values were 100% and 92.68%, respectively when only NCC-negative and also NCC-positive sera were included (Table 2). The AUC for the r2B2t antigen was 0.717 (**Fig 2B**).

### ELISA assays

Both commercially available ELISA tests, Novalisa and Ridascreen, generated a number of grey zone/equivocal results, as defined by the manufacturers’ instructions: for sera from patients with CE, 15 equivocal results were obtained with the Novalisa assay and 10 with the Ridascreen assay. In the case of sera from patients with NCC, there were 7 equivocal results with the Novalisa assay and 14 with the Ridascreen assay. Table 3 summarizes the total number of tested sera and samples with equivocal results. The AUC values for Novalisa and Ridascreen were 0.619 and 0.760, respectively.

**Table 3:** Equivocal results.

		Equivocal results 1		Equivocal results 2	
	Original N	rT24H	Novalisa	r2B2t	Ridascreen
CE	151	0	15	0	10
NCC	126	0	7	0	14

N: number of sera; CE: cystic echinococcosis; NCC: neurocysticercosis

Table 4 summarizes overall and group-specific specificity and sensitivity values, based on samples characteristics when equivocal ELISA tests results were omitted from the analysis and Table 5 when equivocal ELISA results were considered positive or negative.

**Table 4:** Diagnostic accuracy of rT24H- and r2B2t- MBA, and Novalisa, Ridascreen ELISA assays when excluding equivocal ELISA results from the analysis.

		Recombinant antigen T24H					NOVALISA				
Sera categories	N*	(+/-)	Sensitivity (%)	95% CI	Specificity (%)	95% CI	(+/-)	Sensitivity (%)	95% CI	Specificity (%)	95% CI
**Positive NCC (set 1)**	**107**	**62/45**	**57.94**	**48.12–67.76**	-	-	**45/62**	**42.06**	**32.24–51.88**	-	-
Active NCC	38	28/10	73.68	58.37–89	-	-	21/17	55.26	38.14–72.39	-	-
Inactive NCC	55	22/33	40	26.14–53.86	-	-	14/41	25.45	13.03–37.88	-	-
**Negative** **(sets 3 and 2)**	**208**	**19/189**	**-**	-	**90.87**	**86.71–95.02**	**51/157**	-	-	**75.48**	**69.39–81.57**
Healthy (set 3)	79	0/79	-	-	100	99.37–100	1/78	-	-	98.73	95.64–100
CE (set 2)	129	19/110	-	-	85.27	78.77–91.77	50/79	-	-	61.24	52.45–70.04
		Recombinant antigen 2B2t					RIDASCREEN				
Sera categories	N*	(+/-)	Sensitivity (%)	95% CI	Specificity (%)	95% CI	(+/-)	Sensitivity (%)	95% CI	Specificity (%)	95% CI
**Positive CE (set 2)**	**129**	**90/39**	**69.77**	**61.45–78.08**	-	-	**70/59**	**54.26**	**45.28–63.25**	-	-
CE1-CE3b	66	52/14	78.79	68.17–89.41	-	-	51/15	77.27	66.4–88.14	-	-
CE4-CE5	63	38/25	60.32	47.44–73.19	-	-	19/44	30.16	18.03–42.29	-	-
**Negative** **(sets 3 and 1)**	**186**	**14/172**	-	-	**92.47**	**88.41–96.53**	**37/149**	-	-	**80.11**	**74.1–86.11**
Healthy (set 3)	79	0/79	-	-	100	99.37–100	1/78	-	-	98.73	95.64–100
NCC (set 1)	107	14/93	-	-	86.92	80.06–93.77	36/71	-	-	66.36	56.94–75.78

* equivocal results obtained by the commercial tests were excluded from the comparison.

**Table 5:** Diagnostic accuracy of rT24H- and r2B2t- MBA, and Novalisa, Ridascreen ELISA assay when ELISA equivocal results were considered as positive or negative.

		Recombinant antigen T24H					NOVALISA equivocal as positive					NOVALISA equivocal as negative				
Sera categories	N	(+/-)	S(%)	95% CI	Sp (%)	95% CI	(+/-)	S (%)	95% CI	Sp (%)	95% CI	(+/-)	S(%)	95% CI	Sp (%)	95% CI
**Positive NCC** **(set 1)**	**126**	**80/46**	**63.49**	**54.69–72.30**	**-**	**-**	**58/68**	**46.03**	**36.93–55.13**	**-**	**-**	**51/75**	**40.48**	**31.51–49.44**	**-**	**-**
Active NCC	48	37/11	77.08	64.15–90.02	-	-	29/19	60.42	45.54–75.29	-	-	25/23	52.08	36.91–67.26	-	-
Inactive NCC	63	30/33	47.62	34.49–60.75	-	-	18/45	28.57	16.62–40.52	-	-	15/48	23.81	12.50–35.12	-	-
**Negative** **(sets 3 and 2)**	**230**	**20/210**	**-**	**-**	**91.3**	**87.45–95.16**	**68/132**	**-**	**-**	**70.43**	**64.32–76.55**	**53/177**	**-**	**-**	**76.96**	**71.30–82.62**
Set 3	79	0/79	-	-	100	99.37–100	1/79	-	-	98.73	95.64–100	1/78	-	-	98.73	95.64–100
CE (set 2)	151	20/131	-	-	86.75	81.02–92.49	67/84	-	-	55.63	47.37–63.88	52/99	-	-	65.56	57.65–73.47
		Recombinant antigen 2B2t					RIDASCREEN equivocal as positive					RIDASCREEN equivocal as negative				
Sera categories	N	(+/-)	S(%)	95% CI	Sp (%)	95% CI	(+/-)	S (%)	95% CI	Sp (%)	95% CI	(+/-)	S (%)	95% CI	Sp (%)	95% CI
**Positive CE** **(set 2)**	**151**	**104/47**	**68.87**	**61.16–76.59**	**-**	**-**	**87/64**	**57.62**	**49.40–65.83**	**-**	**-**	**77/74**	**50.99**	**42.69–59.30**	**-**	**-**
CE1-CE3b	77	59/18	76.62	66.52–86.73	-	-	60/17	77.92	68.01–87.84	-	-	56/21	72.73	62.13–83.32	-	-
CE4-CE5	74	45/29	60.81	49.01–72.61	-	-	27/47	36.49	24.84–48.13	-	-	21/53	28.38	17.43–39.33	-	-
**Negative** **(sets 3 and 1)**	**205**	**15/190**	**-**	**-**	**92.68**	**88.87–96.49**	**53/152**	**-**	**-**	**74.15**	**67.91–80.38**	**39/166**	**-**	**-**	**80.98**	**75.36–86.59**
Set 3	79	0/79	-	-	100	99.37–100	1/79	-	-	98.73	95.64–100	1/79	-	-	98.73	95.64–100
NCC (set 1)	126	15/111	-	-	88.1	82.04–94.15	52/74	-	-	58.73	49.74–67.72	38/88	-	-	69.84	61.43–78.25

CE: Cystic echinococcosis; NCC: Neurocysticercosis; N: number of sera; CI: confidence interval, S: sensitivity, Sp: specificity.

When excluding equivocal results, for the Novalisa test the sensitivity value for NCC diagnosis was 42.06% and specificity, when considering sera from CE-negative and CE-positive patients, was 75.48%. In the case of the Ridascreen assay, a sensitivity of 54.26% for CE diagnosis and a specificity (including sera from NCC-negative and NCC-positive patients) of 80.11% were obtained (Table 4). The T24H-MBA had a sensitivity for NCC of 57.94%, and a total specificity (including sera from CE-negative and CE- positive patients) of 90.87%. The 2B2t-MBA had a sensitivity of 69.77% for the diagnosis of CE, while total specificity was 92.47% (Table 4). Recombinant antigens with MBA improved the accuracy of the diagnosis compared with commercially available ELISA tests also when including sera having equivocal results on the commercial ELISAs, both when equivocal results were considered positive and negative (Table 5).

### Comparison ROC curves between the MBA and commercially available tests

The diagnostic accuracy of the recombinant antigens tested with the MBA was better than that obtained with the commercial ELISA kits evaluated in parallel in this study. We therefore decided to analyze the ROC curve performed with ELISA-SI data, instead of using of the threshold values established by the manufacturers, to evaluate if a different cut-off of the ELISA assays could achieve better performances. Comparative ROC curves are shown in Fig 2 for the tests used in the diagnosis of each pathology (Novalisa versus T24H, for NCC; Ridascreen versus 2B2t, for CE). A significant difference was found between the MBA-rT24H and the Novalisa commercial test (Chi-square; P <0.001). In the case of Ridascreen, a change in the threshold would lead to an improvement in the values of the expected diagnostic accuracy, although not statistically significant. It could therefore be suggested a modification in the recommended cut-off value (0.6 instead of 1.1) to improve the accuracy of the test if it is going to be used in different conditions to the ones evaluated by the manufactures.

## Discussion

In this study, we standardized a multiplex serodiagnostic method for NCC and CE based on the MBA-platform and recombinant antigens. The use of the 2B2t and T24H recombinant antigens (each previously evaluated for individual infections and having shown promising results) with the MBA technology allows testing both parasitic infections in a single well. Our test enables evaluating a large number of samples with better sensitivity than that of two individual commercially available ELISA tests widely used in Europe for CE and NCC, and with a remarkable reduction of the cross-reactivity usually seen in serological diagnosis between these two conditions [33].

NCC and CE are two neglected diseases with wide geographical distribution that overlap in many Asian, South American, and African regions [34,35]. They are conditions with high morbidity associated to large economic costs for their diagnosis, treatment, and follow-up. Their diagnosis is based on the results of imaging studies and complemented by serological tests in case of inconclusive imaging characteristics. Both diseases are potentially eliminable as public health concerns. Currently, no national *T*. *solium* control programs have been established in endemic areas [36] The WHO 2030 roadmap for NCC identified as a goal the implementation of control measures in hyperendemic areas of 17 endemic countries, however, one of the main difficulties to overcome is that the extent of those hyperendemic areas has not yet been delineated, partly because many limitations exist with current (serological and other) diagnostic methods [21]. Control programs implementation, monitoring and evaluation depend on the availability of sensitive, specific, and reproducible techniques capable of evaluating a large number of samples at affordable costs. In this regard, currently available immunological diagnostic techniques should be improved to facilitate the identification of active transmission hotspots and determine seroprevalence. To this, serological diagnostic assays need to reach improved sensitivity and specificity, avoiding cross-reactivity with other phylogenetically related parasites such as occurs between *T*. *solium* and *E*. *granulosus* [33].

The multiplex bead-based technology derives from classical antibody detection techniques, such as ELISA; it has higher reproducibility and allows the analysis of multiple antigens simultaneously in a single well, with consequent saving of time and sample volume [37,38]. This innovative, versatile technique, allowing the combination of different recombinant antigens targeting multiple pathogens, is becoming a promising tool for joint serological surveillance of diseases of public health importance. For example, Fuji *et al*. [39] performed a joint serological surveillance of six infectious diseases using eight different recombinant antigens (*Entamoeba histolytica* [C-IgL], *Leishmania donovani* [KRP42], *Toxoplasma gondii* [SAG1], *Wuchereria bancrofti* [SXP1], HIV [gag, gp120, and gp41], and *Vibrio cholerae* [cholera toxin]). Furthermore, assessing multiple antigens of agent(s) causing one disease is also possible, as it has been done for malaria, for which antibodies against antigens of different *Plasmodium* species (PfMSP1, PvMSP1, PmMSP1, PfCSP, PfAMA1, PfLSA1, PfGLURP-R0, PfHRP2), have been detected in a single step [40].

The success of MBA technology depends on the diagnostic quality of the used recombinant antigens. For CC, rT24H was selected because it had already shown to provide results in MBA similar to those obtained with the lentil-lectin glycoprotein enzyme-linked immunoelectrotransfer blot test (LLGP EITB), considered the gold standard for the diagnosis of CC in serum [28]. The T24H antigen is a hydrophilic extracellular domain with 92 amino acid residues of *T*. *solium*, T24 tetraspanin, a protein consisting of four transmembrane domains that correspond to the 24 and 42 kDa native antigen bands seen in the LLGP EITB [41]. For the diagnosis of CE, the selected r2B2t recombinant antigen consists of two tandem repeats of the rB2t antigen, which is a C-terminal truncation of *E*. *granulosus* AgB2. r2B2t was chosen for its proven diagnostic value in ELISA [24].

MBA with recombinant antigens has not been used to date for the diagnosis of CE and we highlight the importance of applying this technology in conjunction with a *T*. *solium*-specific recombinant antigens, as both parasites have a close phylogenetic relationship, which is at the basis of antibody cross-reactivity [42]. The results of this study are encouraging. Notwithstanding 73% of the sequence of antigen T24H is shared with two *E*. *granulosus* sequences (BI244014 and BF643023) [41], the percentage of cross-reactive sera from a large number of patients with CE tested with the rT24H was low. Similarly low was the cross-reactivity of sera from patients with NCC when tested on the r2B2t antigen. In fact, cross-reactivity was similar or even lower than that described for other *T*. *solium* recombinant antigens with diagnostic usefulness, e.g., AgTs8B [28,43], and much lower than that showed with crude extracts and excretory/secretory products commonly used in commercially available systems. In this study, with commercial ELISA tests, about one third of the samples showed cross-reactivity.

When sera from uninfected subjects (i.e. healthy + CE-infected for T24H-MBA, and healthy + NCC-infected for 2B2t-MBA) were tested, specificity was similar to those of other studies investigating these antigens (~ 92% in both cases) [17,23,28]. Besides, 100% specificity was obtained when we evaluated sera from patients with other neurological diseases.

The sensitivity of rT24H for NCC was significantly higher than that of the commercial Novalisa test, but lower than what reported in other studies. Sensitivity of seroassays is known to be influenced by the localization of cysticerci, as well as by their number and viability [15]. Circulating antibodies are more easily detected in patients with multiple viable lesions or with extraparenchymal NCC, while a marked drop in sensitivity is observed in patients with a single brain lesion or with inactive cysticerci. In this work, we could only stratify our data by viability, and the serum panel was composed by a larger number of samples from patients with inactive compared to active cysticerci, which could explain the lower sensitivity obtained.

Similarly, the sensitivity of seroassays for CE depends on multiple factors, including cyst stage, number, size, organ affected and previous treatment [44,45]. Grouping of the sera as per the available clinical information on cyst stage, we observed, in accordance with previous reports [45], a greater sensitivity in patients with cysts in CE3a and CE3b stages; however, analysis by individual cyst stage, and in particular of the performance of the test on early, unilocular CE1 cysts, was not possible because the number of sera samples from patients in this early CE stage was very small.

The multiplex bead-based assay including the T24H recombinant antigen has recently been applied on sera obtained from whole blood samples collected on filter paper, showing its potential to be used on samples collected from underserved areas and to be used in the context of control programs [46]. The inclusion in the multiplex bead-based technology of the 2B2t antigen together with the T24H may allow its inclusion in CC epidemiological studies, having not only good sensitivity but also low cross-reactivity with CE and the ability to detect false-positive results due to cross-reactivity to the presence of this target in the same assay. As for CE, the use of seroassays is currently not recommended in the context of epidemiological surveys due to their inadequate performance for this purpose and especially for the diagnosis of early infection and of infection in extra-hepatic organs [25,26,47]. As new “-omic” technologies are generating information on putative antigens, MBA technology could be used to jointly evaluate the diagnostic utility of these new candidates and improve current limitations of CE serological methods, such as lack of markers of early infection, of cyst viability, etc. In addition, other recombinant antigens such as Em2, Em18, EM13 previously tested in the serological diagnosis of alveolar echinococcosis (AE) [16] caused by *Echinococcus multilocularis* and highly cross-reactive with *E*. *granulosus* could be evaluated by MBA in the future. Unfortunately, AE was not included in the present work due to unavailability of sera, representing a limitation of this study.

In conclusion, the MBA technique showed a non-optimal, but improved diagnostic accuracy compared to commercialized ELISA tests for the separate diagnosis of the CC and CE. The use of the MBA technique will allow managing a large number of samples, providing an effective and rapid support to serosurvey studies to detect CC hot-spots where control programs might be implemented. Moreover, this technology has the potential to incorporate new antigens for the simultaneous diagnosis of a larger number of pathologies or improve current serodiagnostic characteristics in the future.
